# Modeling the influence of optic flow on grid cell firing in the absence of other cues^1^

**DOI:** 10.1007/s10827-012-0396-6

**Published:** 2012-05-05

**Authors:** Florian Raudies, Ennio Mingolla, Michael E. Hasselmo

**Affiliations:** 1 Center of Excellence for Learning in Education, Science, and Technology (CELEST), Boston University, 677 Beacon Street, Boston, MA 02215 USA; 2 Center for Computational Neuroscience and Neural Technology, Boston University, 677 Beacon Street, Boston, MA 02215 USA; 3 Department of Psychology, Boston University, 2 Cummington Street, Boston, MA 02215 USA

**Keywords:** Optic flow, Grid cell firing, Entorhinal cortex, Spherical camera, Visual image motion, Gaussian noise model, Self-motion

## Abstract

Information from the vestibular, sensorimotor, or visual systems can affect the firing of grid cells recorded in entorhinal cortex of rats. Optic flow provides information about the rat’s linear and rotational velocity and, thus, could influence the firing pattern of grid cells. To investigate this possible link, we model parts of the rat’s visual system and analyze their capability in estimating linear and rotational velocity. In our model a rat is simulated to move along trajectories recorded from rat’s foraging on a circular ground platform. Thus, we preserve the intrinsic statistics of real rats’ movements. Visual image motion is analytically computed for a spherical camera model and superimposed with noise in order to model the optic flow that would be available to the rat. This optic flow is fed into a template model to estimate the rat’s linear and rotational velocities, which in turn are fed into an oscillatory interference model of grid cell firing. Grid scores are reported while altering the flow noise, tilt angle of the optical axis with respect to the ground, the number of flow templates, and the frequency used in the oscillatory interference model. Activity patterns are compatible with those of grid cells, suggesting that optic flow can contribute to their firing.

## Optic flow as one cue for self-positioning and self-orientation

Visual input from optic flow provides information about self-motion and, thus, could be one cue for the rat’s grid cell system. To characterize the quality of an optic flow input, we suggest a model for optic flow of the rat’s visual system that estimates linear and rotational velocity. These estimated velocities are fed into an oscillatory interference model for grid cell firing. Firing patterns generated by the concatenation of these two models, the template model and oscillatory interference model are evaluated using a grid score measure.

In this modeling effort we exclusively study the optic flow cue and exclude any contributions from other systems for purposes of analysis. However, we are aware of other cues such as landmarks, vestibular, or sensorimotor signals that might have strong influence on grid cells (Hafting et al. 2005; Barry et al. 2007). Thus, this study serves as a foundation for considering more complex, multi-cue paradigms.

### Does the rat’s vision allow for the processing of optic flow?

Low-level processing mechanisms for visual motion exist in rat’s cortex. The vast majority of neurons in primary visual cortex respond to visual image motion (95 %) and the rest to flashing stimuli (Burne et al. 1984). Motion sensitive neurons show tuning for orientation, spatial frequency, and temporal frequency of a presented grating. The optimal stimulus velocity varies between 10 °/s to 250 °/s, and some neurons are selective to velocities of 700 °/s (Girman et al. 1999). Although a hierarchy of visual processing similar to the one in monkey cortex (Fellman and Van Essen 1991) has been pointed out based on the morphology (Coogan and Burkhalter 1993), the functional mapping of these anatomically identified areas is largely unknown.

Visual cues clearly contribute to the spatial response properties of neurons in the entorhinal cortex and hippocampus, including the responses of grid cells (Hafting et al. 2005; Barry et al. 2007), boundary vector cells (Solstad et al. 2008; Lever et al. 2009), head direction cells (Taube et al. 1990) and place cells (O'Keefe and Nadel 1978; Muller and Kubie 1987). Visual cues clearly influence the firing of these neurons, as rotations of a white cue card on a circular barrier causes rotations of the firing location of place cells (Muller and Kubie 1987) and grid cells (Hafting et al. 2005) and the angle of firing of head direction cells (Taube et al. 1990). Compression or expansion of the environment by moving barriers causes compression or expansion of the firing fields of place cells (O'Keefe and Burgess 1996) and grid cells (Barry et al. 2007). These data demonstrate the important role of visual input as one cue for generating spatial responses of grid cells. But visual input is not the only factor influencing firing, as grid cells and place cells can continue to fire in the same location in the darkness (O'Keefe and Nadel 1978; Hafting et al. 2005), and on the basis of self-motion cues without visual input (Kinkhabwala et al. 2011), so visual input is just one out of many possible influences on the firing of these cells. Experimental data has not yet indicated what type of visual input is essential. Detection of the distance and angle of barriers (Barry et al. 2007) could involve learning of features and landmarks, but could also leverage optic flow. Modeling the potential mechanism for the influence of optic flow on the neural responses of grid cells provides a means to test the possible validity of optic flow as a cue contributing to the firing location of these cells.

In our proposed model for rat’s processing of optic flow we assume areas of higher-level motion selectivity for the detection of self-motion from optic flow, similar to those found in macaque monkey’s area MST (Duffy and Wurtz 1995; Duffy 1998; Graziano et al. 1994). Although visual processing for rats is different—major differences occur in visual acuity and space variant vision—we assume that these mechanisms of large field motion pattern detection are not critical with respect to these properties provided by monkey’s cortex. In other words large field motion pattern can be detected also with a uniform sampling and low visual acuity, in our study 40 × 20 samples, and we do not use these properties of high visual acuity and space variant vision in our simulations.

### Optic flow encodes information about directional linear and rotational velocities, relative depth, and time-to-contact

Brightness variations can occur due to object motion, background motion, or changes in lighting. Optic flow denotes the motion of brightness patterns in the image (Horn 1986, p. 278 ff.). To describe optic flow, models of visual image motion have been developed that denote the 3D motion of points projected into the image. Most models use a pinhole camera (Longuet-Higgins and Prazdny 1980; Kanatani 1993) or a spherical camera (Rieger 1983; Fermüller and Aloimonos 1995; Calow et al. 2004) and in both cases the center of rotation is located in the nodal point of the projection. Along with the camera model, the translation and rotation in 3D of the sensor is described by an instantaneous or differential motion model (Goldstein et al. 2001) which neglects higher order temporal changes like accelerations. As simplification we assume here all temporal changes of brightness, optic flow, and visual image motion are the same vector field.

In general optic flow contains information of the sensor’s linear and rotational 3D velocities, and relative depth values of objects in the environment. Several algorithms have been proposed for the estimation of linear and rotational velocities from optic flow using models of visual image motion. Different objective functions, e.g. the Euclidean difference between sensed optic flow and modeled visual image motion, have been used to formulate linear (Kanatani 1993; Heeger and Jepson 1992) and non-linear optimization problems (Bruss and Horn 1983; Zhang and Tomasi 1999). Specific objective functions have been directly evaluated by using biologically motivated neural networks (Perrone 1992; Perrone and Stone 1994; Lappe and Rauschecker 1993; Lappe 1998).

With linear velocity and relative depth, both estimated from optic flow, time-to-contact between the sensor and approaching surfaces can be computed (Lee 1976). For instance, plummeting gannets estimate their time to contact with the water surface from radial expanding optic flow, to close their wings at the right time before diving into the water (Lee and Reddish 1981).

Information about linear and rotational velocities estimated from optic flow could contribute to the firing of head direction and place cells in rats. By temporally integrating rotational velocities the head’s direction relative to some fixed direction (e.g. geographical north) can be computed. Thus, this integrated velocity signal could directly serve as input to the head direction cell system. Experiments support such integration. For instance, if visual motion and landmark cues contradict each other, often head direction cells average signals from both cues (Blair and Sharp 1996). Place cells are influenced by visual motion cues as well. Rats in a cylindrical apparatus with textured walls and textured floor which can be independently rotated show a more reliable update of place field firing if walls and floor have a compatible rotation. Thus, place cell firing is influenced by visual and vestibular signals (Sharp et al. 1995). Border cells (Solstad et al. 2008) could integrate time to contact information from walls that give rise to optic flow of high velocity since the length of flow vectors for translational motion is inversely related to distance. Furthermore, integration of linear velocity and rotational velocity provides a position signal that could serve as an input to the grid cell firing system. However, this temporal integration or path integration typically encounters the problem of error summation. Small errors accumulate over the course of integrating thousands of steps and can potentially lead to large deflections in the calculated animal’s spatial position. In order to study this problem of error summation and its influence on grid cell firing, we propose a model for optic flow in rats and a mechanism for self-motion detection and test its ability to generate grid cell firing.

## Methods

A model for optic flow processing in rats and its influence on grid cell firing is developed in the following steps. Linear and rotational velocities are extracted from recorded rat trajectories that are pre-processed to model the rat’s body motion (Section 2.1). Visual image motion is simulated moving a spherical camera along these pre-processed trajectories (Section 2.2). In order to estimate the linear velocity the visual image motion model is constrained to points sampled from a horizontal plane (Section 2.3). Optic flow is modeled by adding Gaussian noise to the visual image motion which accounts for errors that would occur if flow was estimated from spatio-temporal varying patterns of structured light (Section 2.4). A template model estimates linear and rotational velocity from optic flow (Section 2.5). Finally, these estimated velocities are integrated into an oscillatory interference model to study grid cell firing (Section 2.6).

### Simulating the rat’s locomotion by linear and rotational velocities

Typically in experiments only the head movement of rats is recorded and not their body movement or eye movement. This recorded data only allows an approximate calculation of the rat’s retinal flow due to body motion, which is based on the assumption that the monocular camera that models the rat’s entire field of view is forward directed and tangent to the recorded trajectory. This assumption leads to rotational velocities of nearly 360 ° between two sample points, leading to a rotational velocity of 360 ° · 50 Hz = 18,000 °/s, which seems unrealistically high, especially as visual motion sensitive cells in rats V1 are selective to speeds only up to 700 °/s (Girman et al. 1999). Looking at the behavior, these high rotational velocities occur due to head/sensor movements that are independent of body movements. Here we do not account for either of these movements. In addition jitter occurs in the recorded position. For these reasons we pre-processed all recorded rat’s trajectories with an iterative method that is based on three rules. First, data points are added by linear interpolation, if the rotation between two samples is above 90 ° or 4500 °/s for a 50 Hz sampling rate. Second, data points are removed if the displacement between two points is below a threshold of 0.05 cm or 2.5 cm/s for a 50 Hz sampling rate. Speeds below 2.5 cm/s are considered to indicate that the rat is stationary, as data shows that the distribution of recorded speeds peaks around 10 cm/s and tails off toward 50 cm/s. Third, data points are added by linear interpolation between samples that lead to linear velocities above 60 cm/sec. This pre-processing changes less than 17 % of the original data points, whereas Kalman filtering of the trajectories would change all data points. After pre-processing we assume that the trajectory is close to the actual body motion of the rat.

The rat’s enclosure is modeled by a circular ground platform that has a radius of 100 cm. This radius is 15 cm larger than the cage’s radius used in the experiment. We added an annulus of 15 cm in the simulations to allow for sensing visual motion in cases where the camera is close to the boundary and the optical axis points toward the boundary. The recorded positional data can be modeled by two degrees of freedom: A linear velocity parallel to the ground and a rotational velocity around the axis normal to the ground. In our simulation the camera is shifted and rotated, and analytical flow is calculated using a depth map computed by ray-tracing.

**Fig. 1:**
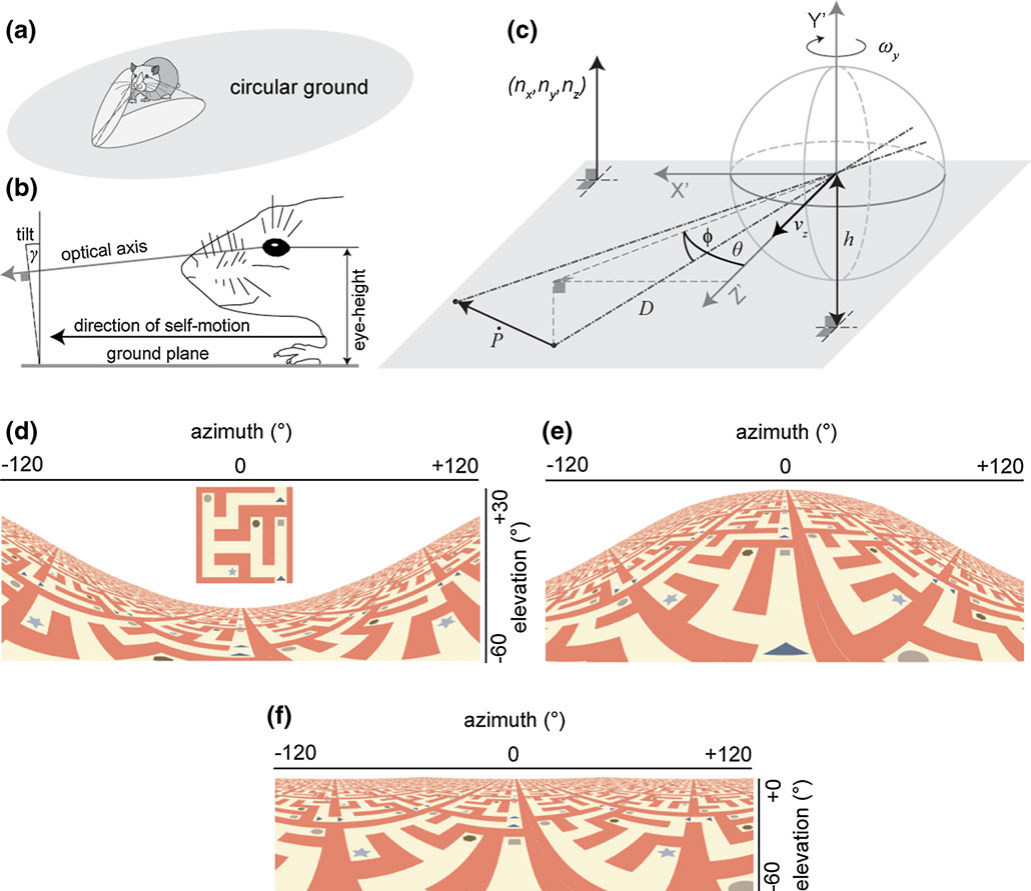
Our model for visual image motion of a rat. (**a**) A virtual rat is simulated running along recorded trajectories (Hafting et al. 2005). Optic flow is modeled for the overall visual field, which includes left and right hemisphere. (**b**) Shows the eye-height *h* and tilt angle *γ* of the optical axis with respect to the ground; adapted and redrawn from Adams and Forrester (1968), their Figure 6. (**c**) Visual image motion is generated for sample points *P* on the ground as the rat is moving parallel to the ground or rotating around an axis normal to the ground. This drawing is for *γ* = 0 ° tilt and, thus, the linear velocity is along the optical axis and the rotational velocity around the y-axis (yaw). (**d**-**f**) Projections of a regular labyrinth texture (inset in d) tiled on the ground floor within ±120 ° azimuth and ±60 ° elevation using the spherical camera model. The nodal point of the spherical camera is *h* = 3.5 cm above the ground plane 120 x 120 cm large and is placed in the center of the plane. Tilt angle varies, in (**d**) it is *γ* = -30 °, in (**e**) *γ* = 30 °, and in (**f**) *γ* = 0 °. All three images were rendered with Persistence of Vision Pty. Ltd. (2004). Note that the labyrinth texture does not coincide with some maze-task but has only been chosen for visualization purposes. Instead the simulated rat can freely move on a ground platform

Figure 1(a) illustrates the rat sitting on the ground with its field of views for left and right eye which we model as a single visual field of view excluding binocular vision. The nodal point of the modeled camera is at height *h* above the ground and the y-axis of the camera’s coordinate system has a tilt angle of *γ* with respect to the ground plane (Fig. 1(b)). Positive tilt angles *γ* rotate the camera downward, negative ones upward. To model the field of view we use a spherical camera (Fig. 1(c)). Projections of a labyrinth texture tiled on the ground for this spherical camera are shown in Fig. 1(d)-(f). In the next paragraph we define the projection function and a model of visual image motion for this spherical camera model.

### Modeling the rat’s visual image motion using a spherical camera

Why do we use a spherical camera to model the visual image motion sensed by rats? First, rat eyeballs are more similar to spheres than a plane, as used in a pinhole camera. Second, with a spherical camera we can model a field of view larger than 180 °, impossible with a single pinhole camera.

Visual image motion for a spherical camera with its center of rotation, nodal point, and center of the sphere in the same point, is described in spherical coordinates by (Rieger 1983; Calow et al. 2004)^1^:

1 $$ \begin{array}{*{20}{c}}  {\left( {\begin{array}{*{20}{c}}   {\dot{\theta }} \hfill  \\   {\dot{\phi }} \hfill  \\  \end{array} } \right){\text{ }} = \frac{1}{D}\left( {\begin{array}{*{20}{c}}   { - \frac{{\cos (\theta )}}{{\cos (\phi )}}} & 0 & {\frac{{\sin (\theta )}}{{\cos (\phi )}}}  \\   {\sin (\theta )\cdot \sin (\phi )} & { - \cos (\phi )} & {\cos (\theta )\cdot \sin (\phi )}  \\  \end{array} } \right)\left( {\left( {\begin{array}{*{20}{c}}   1 & 0 & 0  \\   0 & {\cos \gamma } & { - \sin \gamma }  \\   0 & {\sin \gamma } & {\cos \gamma }  \\  \end{array} } \right)\left( {\begin{array}{*{20}{c}}   {{{v}_{x}}}  \\   {{{v}_{y}}}  \\   {{{v}_{z}}}  \\  \end{array} } \right)} \right)} \\  { + \left( {\begin{array}{*{20}{c}}   {\frac{{\sin (\theta )\cdot \sin (\phi )}}{{\cos (\phi )}}} & { - 1} & {\frac{{\cos (\theta )\cdot \sin (\phi )}}{{\cos (\phi )}}}  \\   {\cos (\theta )} & 0 & { - \sin (\theta )}  \\  \end{array} } \right)\left( {\left( {\begin{array}{*{20}{c}}   1 & 0 & 0  \\   0 & {\cos \gamma } & { - \sin \gamma }  \\   0 & {\sin \gamma } & {\cos \gamma }  \\  \end{array} } \right)\left( {\begin{array}{*{20}{c}}   {{{\omega }_{x}}}  \\   {{{\omega }_{y}}}  \\   {{{\omega }_{z}}}  \\  \end{array} } \right)} \right)} \\  \end{array} , $$

where *θ* denotes the azimuth angle, *ϕ* the elevation angle, and *γ* the tilt angle between optical axis and self-motion vector that is always parallel to the ground (see Fig. 1(b)). Azimuth is measured in the xz-plane from the z-axis that points forward. Elevation is measured from z'-axis in the yz'-plane where z' denotes the z-axis rotated by the azimuth angle. We use a left-handed coordinate system. In this system the z-axis points forward, the y-axis points to the top and the x-axis points to the right (see also Fig. 1(c)). The 3D linear velocity $\vec{v} = {({v_x},\;{v_y},\;{v_z})^t}$ and the 3D rotational velocity $\vec{\omega } = {({\omega_x},\;{\omega_y},\;{\omega_z})^t}$ cause temporal differentials for azimuth ${\mathop \theta \limits^. }$ and elevation ${\mathop \phi \limits^. }$ assuming a differential motion model that neglects higher order temporal differences, like accelerations (Goldstein et al. 2001; Longuet-Higgins and Prazdny 1980). The super-index ‘*t*’ denotes the vector-transpose. Furthermore, $D = \sqrt {{{X^2} + {Y^2} + {Z^2}}}$ denotes the absolute distance for a point *P* = (*X*, *Y*, *Z*) in Cartesian coordinates.

This model for visual image motion has several properties. First, translational and rotational components are linearly superimposed. Second, only the translational component depends on distance *D*. Third, this depth dependence is reciprocal. Thus, farther points have lower velocities than closer points in terms of temporal changes of azimuth and elevation. Fourth, the image motion that is denoted in differential changes of azimuth and elevation angles is independent of the radius of the sphere that models the rat’s eyeball.

Based on the data we assume that the rat is moving tangent to the recorded trajectory in the 2D plane. This simplifies the model’s linear velocity to be $\vec{v} = {(0,\;0,\;{v_z})^t}$ and the rotational velocity to be $\vec{\omega } = {(0,\;{\omega_y},\;0)^t}$. Including these constraints Eq. (1) reduces to a model of visual image motion for curvilinear motion:

2 $$ \begin{array}{*{20}{c}}  {\left( {\begin{array}{*{20}{c}}   {\dot{\theta }} \hfill  \\   {\dot{\phi }} \hfill  \\  \end{array} } \right) = \frac{{{{v}_{z}}}}{D}\left( {\begin{array}{*{20}{c}}   {\frac{{\sin (\theta )}}{{\cos (\phi )}}\cdot \cos (\gamma )}  \\   {\cos (\phi )\cdot \sin (\gamma ) + \cos (\theta )\cdot \sin (\phi )\cdot \cos (\gamma )}  \\  \end{array} } \right)} \\  { + {{\omega }_{y}}\left( {\begin{array}{*{20}{c}}   { - \cos (\gamma ) + \frac{{\cos (\theta )\cdot \sin (\phi )}}{{\cos \phi }}\cdot \sin (\gamma )}  \\   { - \sin (\theta )\cdot \sin (\gamma )}  \\  \end{array} } \right)} \\  \end{array} . $$

In this model self-motions are restricted to the rotational velocity *ω*_*y*_ around an axis normal to the ground and the linear velocity *v*_*z*_ parallel to the ground. If tilt is unequal to zero, the optical axis of the camera system changes while the self-motion vector remains constant.

Since we do not explicitly model both eyes but rather the entire visual field, we assume this field to extend ±120 ° horizontally and ±60 ° vertically and 0 ° horizontally is aligned with the optical axis (z-axis). Retinal input projects to lateral geniculate nucleus (LGN) in these ranges in rats (Montero et al. 1968).

Absolute linear velocity (speed) and depth appear to be invariant in Eqs. (1) and (2). Any factor multiplied to both variables $\vec{v}$ for Eq. (1) or *v*_*z*_for Eq. (2) and *D* leads to the same spherical image motion. For instance, projected points have the same displacement in angular coordinates either traveling twice as fast or all sample points being two times closer. To resolve this invariance further constraints are required and we provide one solution relevant for rats next.

### Constraining the linear velocity by sampling from a plane of known distance and spatial orientation in 3D

To resolve the invariance between absolute velocity and depth, visual image motion is constrained to sample points from a plane of known distance and 3D orientation. Evidence for such a constraint is provided from a different species, namely frogs, which use “retinal elevation”, the distance of the eyes above ground, to estimate the distance of prey (Collet and Udin 1988). This plane constraints distance values to be

3 $$ D(\theta, \varphi ) = \frac{h}{{{n_x} \cdot \sin (\theta ) \cdot \cos (\varphi ) + {n_y} \cdot \sin (\varphi ) + {n_z} \cdot \cos (\theta ) \cdot \cos (\varphi )}}, $$

where $\vec{n} = {({n_x},{n_y},{n_z})^t}$ is the normal vector of the plane and *h* the plane’s distance measured along the plane’s normal. In our simulations we further constrain the normal vector by tilt angle *γ* between the optical axis and the ground-plane; thus, $\vec{n} = \left( { - \sin \phi \sin \gamma, \;\cos \gamma, - \cos \phi \sin \gamma } \right)$ which depends now also on the allocentric camera or head direction *φ*. This normal vector $\vec{n}$ can be computed, e.g., by using Rodrigues rotation equation and rotating the normal vector (0, 1, 0) around the axis ( − cos *ϕ*, 0, sin *ϕ*). Plugging in both constraints gives the constrained model for visual image motion:

4 $$ \begin{array}{*{20}{c}}   {\mathop{{\overrightarrow \psi  }}\limits^{\cdot }  = \frac{1}{h}\cdot \overrightarrow a \cdot {{v}_{z}} + \overrightarrow b \cdot {{\omega }_{y}}\,{\text{with}}\,\mathop{{\overrightarrow \psi  }}\limits^{\cdot }  = \left( {\begin{array}{*{20}{c}}   {\dot{\theta }} \hfill  \\   {\dot{\phi }} \hfill  \\  \end{array} } \right),} \hfill  \\   {\overrightarrow a  = ( - \sin (\phi )\cdot \sin (\gamma )\cdot \sin (\theta )\cdot \cos (\phi )} \hfill  \\   { + \cos (\gamma )\cdot \sin (\phi ) - \cos (\phi )\cdot \sin (\gamma )\cdot \cos (\theta )\cdot \cos (\phi )),} \hfill  \\   {\cdot \left( {\begin{array}{*{20}{c}}   {\frac{{\sin (\theta )}}{{\cos (\phi )}}\cdot \cos (\gamma )}  \\   {\cos (\phi )\cdot \sin (\gamma ) + \cos (\theta )\cdot \sin (\phi )\cdot \cos (\gamma )}  \\  \end{array} } \right)} \hfill  \\   {{\text{and}}\,\overrightarrow b  = \left( {\begin{array}{*{20}{c}}   { - \cos (\gamma ) + \frac{{\cos (\theta )\cdot \sin (\phi )}}{{\cos \phi }}\cdot \sin (\gamma )}  \\   { - \sin (\theta )\cdot \sin (\gamma )}  \\  \end{array} } \right).} \hfill  \\  \end{array}  $$

**Fig. 2:**
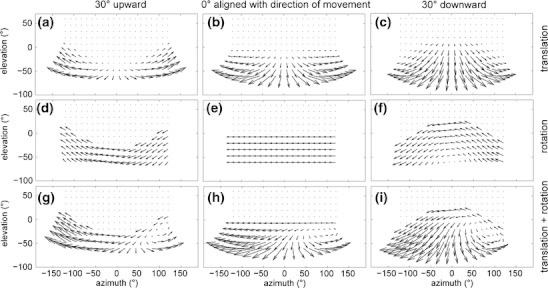
Visual motion fields for a spherical camera model for varying tilt and self-motion defined as a linear speed of *v*_*z*_ = 2 cm/s and/or a rotational velocity of *ω*_*y*_ = 15 °/s. (a-c) Shows motion fields generated by the forward motion, in (**a**) for γ = -30 °, (**b**) for γ = 0 °, and (**c**) for γ = 30 °. (**d**-**f**) Shows motion fields generated by a rotation along an axis orthogonal to the ground. Roll is very dominant in the motion field of (**f**), which is a result of the camera pointing downward while rotating around an axis orthogonal to the ground that is not the vertical axis of the camera. (**g**-**i**) Depict motion fields that are the result of adding motion fields from the first and second row. These are examples of motion fields generated by curvilinear motion. The vanishing point in (**i**) that appears around 90 ° azimuth and -20 ° elevation does not denote the theoretical focus of expansion. Instead it shows the center of motion that is the focus of expansion shifted by the rotational visual motion component. In all plots, the x-axis to y-axis has an aspect ratio of 1:2

Note that this constrained model of visual image motion is not directly accessed by the retina rather it provides a constraint for flow templates that are specific for flow sampled from the ground of known distance. Illustrations of constrained spherical motion flows for forward motion, rotational motion, and their combination are shown in Fig. 2. For the model we assume flow templates to be realized by cortical areas in the rat’s brain that are analogous to the middle temporal area (MT) and medial superior temporal (MST) in the macaque monkey (Graziano et al. 1994; Duffy and Wurtz 1995; Duffy 1998).

### Modeling of the rat’s sensed optic flow by applying Gaussian noise to analytically computed visual image motion

Optic flow is typically estimated from changing brightness values and, therefore, contains errors introduced by the sensing and estimation process. Here, we assume that these errors occur in the sensing of spatial and temporal changes in brightness. These errors are modeled by a Gaussian distribution function, whereas all three variables, two for spatial changes and one for temporal changes, are independent (Simoncelli et al. 1991; their Eq. (7)). Under these assumptions it follows that the distribution of image velocity estimates, using a linear least squares model, is again Gaussian distributed. Thus, in order to model the optic flow that is available to the rat, we assume additive Gaussian noise for the temporal changes of azimuth and elevation angle:

5 $$ \left( {\matrix{{*{20}{c}} {\dot{\tilde{\theta }}} \\ {\dot{\tilde{\varphi }}} \\ } } \right) = \left( {\matrix{{*{20}{c}} {\dot{\theta }} \\ {\dot{\varphi }} \\ } } \right) + \left( {\matrix{{*{20}{c}} {{{\dot{\theta }}_n}} \\ {{{\dot{\varphi }}_n}} \\ } } \right)\,{\text{with}}\,{\dot{\theta }_n},\;{\dot{\varphi }_n}\; \in \;{N_{\mu = 0,\sigma }}_{flow}, $$

where ${N_{\mu = 0,\sigma }}_{flow}$ denotes a normal distribution function with zero mean and *σ*_*flow*_ as standard deviation. Note that $\dot{\varphi }$ and $\dot{\theta }$ denote the model of visual image motion and $\dot{\tilde{\varphi }}$ and $\dot{\tilde{\theta }}$ the modeled optic flow assuming Gaussian noise.

### Estimating linear and rotational velocity using a template model

Linear and rotational velocity for the constrained model of visual image motion from Eq. (4) can be estimated, using the idea of template models: To explicitly evaluate an objective function defined by using neural properties (Perrone 1992; Perrone and Stone 1994). A challenge for template models that estimate self-motion is the 6D parameter space composed of two parameters for the linear velocity direction, one parameter for depth, and three parameters for rotational velocities. All these parameter intervals are sampled explicitly. For instance, for 10 samples in each interval this results in 10^6^ samples for parameters where the sensed flow field has to be compared to all these 10^6^ flow templates. This is a computational expensive approach and, therefore, typically template models use constrained self-motions, e.g. fixating self-motions (Perrone and Stone 1994).

Our constrained model in Eq. (4) has only two parameters: Linear and rotational velocity. Instead of sampling these two dimensions combined, we look for a method of separating the sampling of linear velocity (depth) from that of rotational velocity. Assume that the temporal changes for azimuth and elevation are given by $\dot{\hat{\theta }}$ and $\dot{\hat{\varphi }}$, respectively, which is denoted by the hat-symbol. In the simulation these inputs are from Eq. (5); formally $\dot{\hat{\theta }} = \dot{\tilde{\theta }}$ and $\dot{\hat{\varphi }} = \dot{\tilde{\varphi }}$. To estimate only the linear velocity we multiply the Eq. (4) by ${\vec{b}^\bot }$, that is a vector orthogonal to $\vec{b}$. This multiplication annihilates the dependency on rotational velocity, leaving only the dependency for linear velocity. An objective function *f*_*match*,*v*_ using Gaussian tuning with standard deviation *σ*_*v*_ for the sensed input flow $\dot{\vec{\hat{\psi }}}$ including this constraint is given by

6 $$ {f_{match,v}}({v_{z,j}}) = \frac{1}{n}\sum\limits_{l = 1}^n {\exp \left( { - \frac{{{{(\dot{\vec{\hat{\psi }}}_{_l}^t\vec{b}_l^\bot \cdot h - \vec{a}_l^t\vec{b}_l^\bot \cdot {v_{z,j}})}^2}}}{{2 \cdot {\sigma_v}^2}}} \right)} . $$

In this Eq. (6), ${\vec{a}_l}$ denotes the vector $\vec{a}$ from Eq. (4) that is sampled at locations indexed by *l*. For our sensor layout of the spherical camera model *l* is a linear index for the 20 × 40 grid of elevation and azimuth angles. The orthogonal complement $\vec{b}_l^\bot$ of vector $\vec{b}$ is sampled at the same locations in 20 × 40 grid. The *j*-th linear velocity sample is *v*_*z*,*j*_. Since the model for visual motion is consistent above the entire visual field, tunings from all *n* locations are averaged. This last assumption excludes independently moving objects and deformations that are not induced by self-motion. The objective function *f*_*match*,*ω*_ with the standard deviation *σ*_*ω*_ for rotational velocities is given by using the two constraints of Eq. (4) and the above estimated velocity estimate denoted by $\hat{v}$:

7 $$ {f_{match,\omega }}(\hat{v},{\omega_{y,k}}) = \frac{1}{n}\sum\limits_{l = 1}^n {\exp \left( { - \frac{{{{\left\| {\dot{\vec{\hat{\psi }}}_l - \frac{1}{h}{{\vec{a}}_l} \cdot \hat{v} - {{\vec{b}}_l} \cdot {\omega_{y,k}}} \right\|}^2}}}{{2 \cdot {\sigma_\omega }^2}}} \right)} . $$

Flow templates for all common self-motions are created a priori by using expressions from Eq. (4), like $\vec{a}$ and $\vec{b}$. Every sensed flow field is then compared to all these templates and its match is computed by Eqs. (6) and (7) for linear and rotational velocities, respectively. This method provides match values for each velocity parameter. All match values together define a profile. Next, we will describe methods of estimating velocities from such profiles.

How do we estimate velocities based on the profiles defined by values about the match between template and input flow? One solution is to return the sampled velocity value that corresponds to the maximum match value in the profile, e.g. ${\hat{v}_z} = \arg {\max_j}{f_{match,v}}({v_{z,j}})$. However, estimates of such a method are always restricted to the discrete sampled velocities. An alternative method that overcomes this limitation is a ‘vector’-sum computation. This method sums the product of all velocities—either linear or rotational—multiplied by their corresponding value from the profile of match values and, finally, divides the summed products by the sum of all match values. Such an evaluation of the response profile allows for the interpolation between different velocities. However, this method has a sample bias, if the objective function is not dropping fast enough to zero at the boundaries of the sample interval. As a result a sample bias toward lower or higher velocities can happen at the higher or lower edge of the sample interval, respectively. To avoid sample biases at boundaries we use another method, a local vector summation. This is a combination of the above suggested two methods and restricts the computation of the vector sum to 2 % of sample values centered at the location of the maximum value.

An evaluation of trigonometric functions for the construction of the templates is not directly necessary. Assume that these flow templates with their trigonometric functions from Eq. (4) are ‘hard-wired’ or trained during exposure to visual stimuli. Then coefficients like sin (*θ*), cos (*θ*), … can be expressed as weights for pooling local motion signals over the entire visual space. Furthermore, different tilt angles *γ* and head direction angles *φ* can activate different template representations.

**Fig. 3:**
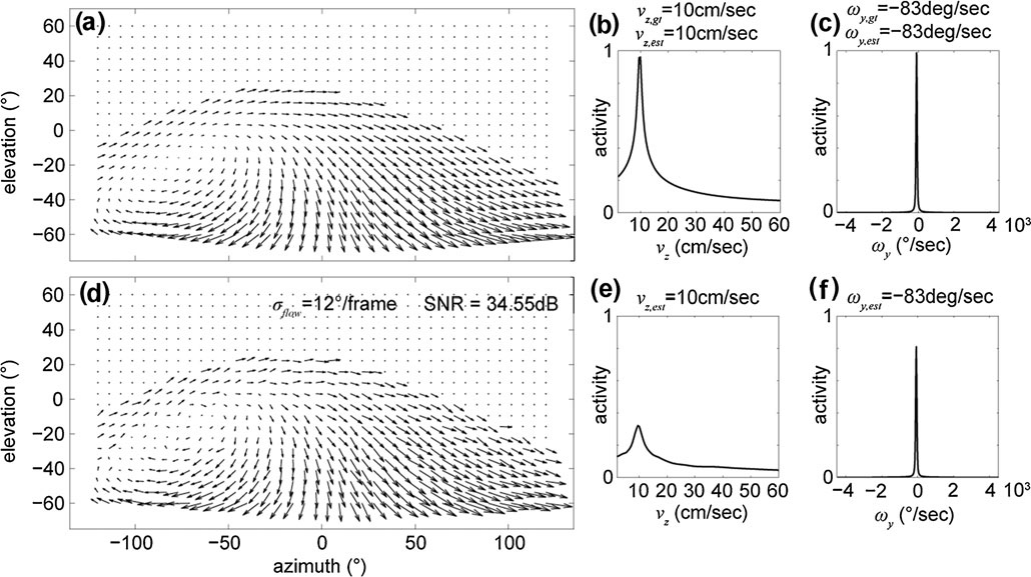
Values for matches of our template model for analytical visual motion and optic flow modeled by visual motion with superimposed Gaussian noise are shown. (**a**) Motion field without noise and a tilt angle of *γ* = 30 °. (**b**) Profile for matches of linear velocities sampled in the range from 2 cm/s to 60 cm/s. (**c**) Match values for rotational velocities within ±4,500 °/s. (**d**) Optic flow defined as the visual motion field from (**a**) with independent Gaussian noise added to each flow component. (**e** and **f**) The profiles of match values for velocities have a decreased peak height but the position of their maximum peak remains. All flow fields are generated taking sample point 10 of the pre-processed rat trajectory ‘Hafting_Fig2c_Trial1’ from Hafting et al. (2005). The aspect ratio between x-axis (azimuth) and y-axis (elevation) is 1:2

Figure 3 shows example flow fields, response profiles, and velocity estimates of our template model. Gaussian noise superimposed to the image motion reduces the peak height, compare Fig. 3(b) and (c) with (e) and (f) but the location of the peak remains. For stronger noise the peak height will be decreased even further, and its position might shift as well, especially if the peak height is falling below the noise floor. In general the peak height could be interpreted as certainty of a match.

The difference in the response profiles for linear and rotational velocity is due to the qualitative difference between flows generated for these. A linear velocity in the interval of 10 cm/s to 60 cm/s introduces slower velocity vectors that are more susceptible to noise than the larger velocity vectors generated by rotational velocities in the range of ±4,500 °/s. These slower vectors for linear velocity are stronger influenced by noise than the larger vectors generated by rotational velocities. As a result, the profile for linear velocity appears “narrower” than that of rotational velocity. Note, this observation depends on our assumption that noise is independent of the length of flow vectors, see also Eq. (5).

A temporal integration of linear and rotational velocity estimates provides an approximation for the rat’s position with respect to some reference. But, these velocities directly cannot explain the regular firing structure of grid cells. Several models (Fuhs and Touretzky 2006; Burgess et al. 2007; Hasselmo 2008; Burak and Fiete 2009) have been proposed to link 2D velocity vectors in the plane to the firing pattern of grid cells. In the next paragraph we review an oscillatory interference model and show how estimates of linear and rotational velocity are integrated into this model.

### Modeling of grid cell firing by temporal integration of estimated velocities within an oscillatory interference model

To solve the problem of dead-reckoning or homing several species rely on path integration (Müller and Wehner 1988). Models for grid-cell firing use this idea and integrate 2D velocity signals. Such a 2D velocity ${\vec{v}_{2D}}$ includes the current orientation *ϕ*(*t*) of the rat’s head in a 2D world and the linear speed *v*_*z*_ *(t)*:

8 $$ {\vec{v}_{2D}}(t) = {v_z}(t) \cdot \left( {\matrix{{*{20}{c}} {\cos \phi (t)} \\ {\sin \phi (t)} \\ } } \right)\,{\text{with}}\,\phi (t) = \int\limits_0^t {{\omega_y}(s)\,ds} $$

The rat’s orientation *ϕ*(*t*) in our case is calculated by temporally integrating all rotational velocities.^2^ Therefore, the rotational velocity has to be estimated besides the linear velocity, since it indicates the orientation of the linear velocity with respect to an external frame of reference, e.g. the middle of the cage. Linear and rotational velocity in Eq. (8) is estimated with the template model described in Section 2.5.

This 2D velocity estimate from Eq. (8) is temporally integrated within the oscillatory interference model (Burgess et al. 2007; Hasselmo 2008):

9 $$ spike(t) = \left\{ {\begin{array}{*{20}{c}}   1 & {\left( {\prod\limits_{{k = 1}}^{3} {\cos (\omega \cdot t) + \cos \left( {\omega \cdot t + \omega \cdot \beta \cdot \int\limits_{0}^{t} {\overrightarrow v _{{2D}}^{t}(s)\cdot {{{\overrightarrow b }}_{k}}\:ds} } \right)} } \right) > \Theta }  \\   0 & {otherwise}  \\  \end{array} } \right.. $$

This model has two oscillations, the somatic oscillation *ω* and the dendritic oscillation $\omega \cdot (t + \beta \cdot \int {...} )$ and the latter one is the somatic oscillation modulated by the integrated velocity signal and by the parameter *β*. It is assumed that the oscillation *ω* is provided by theta rhythm oscillations provided by the medial septum (Brandon et al. 2011). Furthermore, in the model three oscillations interfere at the soma, whereas each oscillation occurs along a basis vector ${\vec{b}_k}$. To form a hexagonal grid with vertices corresponding to high grid cell firing, at least two basis vectors rotated to one another by arbitrary multiples of 60 ° are required. In our implementation we chose the three vectors: ${\vec{b}_1} = {(\cos 0^\circ, \;\sin 0^\circ )^t}$, ${\vec{b}_2} = {(\cos 120^\circ, \;\sin 120^\circ )^t}$, and ${\vec{b}_3} = {(\cos 240^\circ, \;\sin 240^\circ )^t}$. Each vector alone generates a band of firing with an inter-band distance *L* = 1/(*β* ⋅ *f*) because the constructive interference between a purely somatic oscillation *f* = *ω*/(2*π*) and dendritic oscillation *f* + *f* ⋅ *β* results in an oscillation with the overall envelope of frequency *f* + *f* ⋅ *β* – *f* = *f* ⋅ *β*. The overlay of all three dendritic oscillations and bands reaches values above threshold *Θ* in vertices of a hexagonal grid. The hexagonal grid structure originates from the above defined basis vector system. The grid distance is $G = 2/(\sqrt {3} \cdot \beta \cdot f)$. All parameter values of the model are listed in Table 1.

**Table 1:** Parameters and their values set in simulations for the spherical camera model, template model, and oscillatory interference model

Description of parameter	Value
Pre-processing of Simulated trajectories ^a^	
Adds points for rotations above (90 ° · 50 Hz)	4,500 °/s
Removes points for linear velocities below (0.05 cm · 50 Hz)	2.5 cm/s
Adds points for linear velocities above (1.2 cm · 50 Hz)	60 cm/s
Environment of a ground plane	
Radius of the circular ground platform ^b^	100 cm
Number of triangles to represent the ground platform	32
Spherical camera model	
Horizontal field of view	240 °
Vertical field of view	120 °
Number of sample points in vertical dimension ^c^	40
Number of sample points in horizontal dimension ^c^	20
Minimum distance for a sample point	*D*_*min*_ = 0 cm
Maximum distance for a sample point	*D*_*max*_ = 1000 cm
Eye-height above ground	*h* = 3.5 cm
Tilt angle of the optical axis with respect to ground	*γ* = 0 °
Template model	
Standard deviation of the objective function for the linear velocity *v*_*z*_	*σ*_*v*_ = 10 °/s
Standard deviation of the objective function for the rotational velocity *ω*_*y*_	*σ*_*ω*_ = 25 °/s
Interval of rotational velocities	*ω*_*y,k*_ ∈ {±4,500 °/s}
Samples for rotational velocities	*k* = 1…451
Interval for linear velocities	*v*_*z,j*_ ∈ {2…60 cm/s}
Samples for linear velocities	*j* = 1…117
Oscillatory interference model	
Frequency ^d^	*f* = 7.38 Hz
Parameter ^e^	β = 0.00385 s/cm
Angles for basis vectors	φ_1_ = 0 °, φ_2_ = 120 °, φ_3_ = 240 °
Threshold value for spiking	Θ = 1.8

^a^File names of the trajectories included in the simulation are ‘Hafting_Fig2c_Trial1’, ‘Hafting_Fig2c_Trial2’, and ‘rat_10925’ from the Moser lab: http://www.ntnu.no/cbm/gridcell. For the simulations we extracted the maximum subsequence without not-a-numbers

^b^In the simulation the size of the circular ground platform was extended by 15 cm on each side with respect to the original size of a radius of 85 cm. This extension provides sample points at the outmost boundary of 85 cm and allows for picking up optic flow

^c^This refers to an image resolution of 40 × 20 pixels or n = 800. Note that image flow may not be acquired for each pixel because of the tilt angle and simulating an open cage, see Figs. 2 and 3

^d^Frequency *f* has been fitted to subthreshold oscillations of entorhnial cells (Giocomo et al. 2007)

^e^Parameter *β* has been fitted to the measured subthreshold oscillations for neurons and their simulated grid cell spacing (Giocomo et al. 2007)

## Results

Data on rat’s visual input, head-body position, and functions of cortical higher-order visual motion processing is largely unknown. Therefore, we assume parameter ranges for these unknowns that are simulated. The extraction of optic flow from visual input is modeled by providing analytical visual motion for a spherical camera superimposed with additive Gaussian noise. In the first simulation, the standard deviation *σ*_*flow*_ of this Gaussian noise varies between 0 °/frame and 50 °/frame or 0 °/s and 2,500 °/s assuming a temporal sampling of 50 Hz. Since the head’s position of the rat is tracked in a 2D plane the tilt angle *γ* of the head with respect to the ground is not captured by the recording during the experiment. In the second simulation, we assume a range of tilt angles *γ* varying from –45 ° to +45 °. In the third simulation, the number of flow templates varies between 10 and 568. For the fourth simulation we vary the sub-threshold frequency *f* in the oscillatory interference model that changes the grid spacing. Table 2 provides a summary of parameter values in these simulations.

**Table 2:** Parameter values of our model for the simulations

Simulation type	*σ*_*flow*_ (°/frame)	*γ* (°)	Templates	Frequency (Hz)
Flow noise	0…50	0	568	7.38
Tilt angle	25	–45… + 45	568	7.38
Templates	25	0	10…568	7.38
Subthreshold frequency	25	0	568	3.87…7.38

### Error statistics of the estimated linear and rotational velocity

**Fig. 4:**
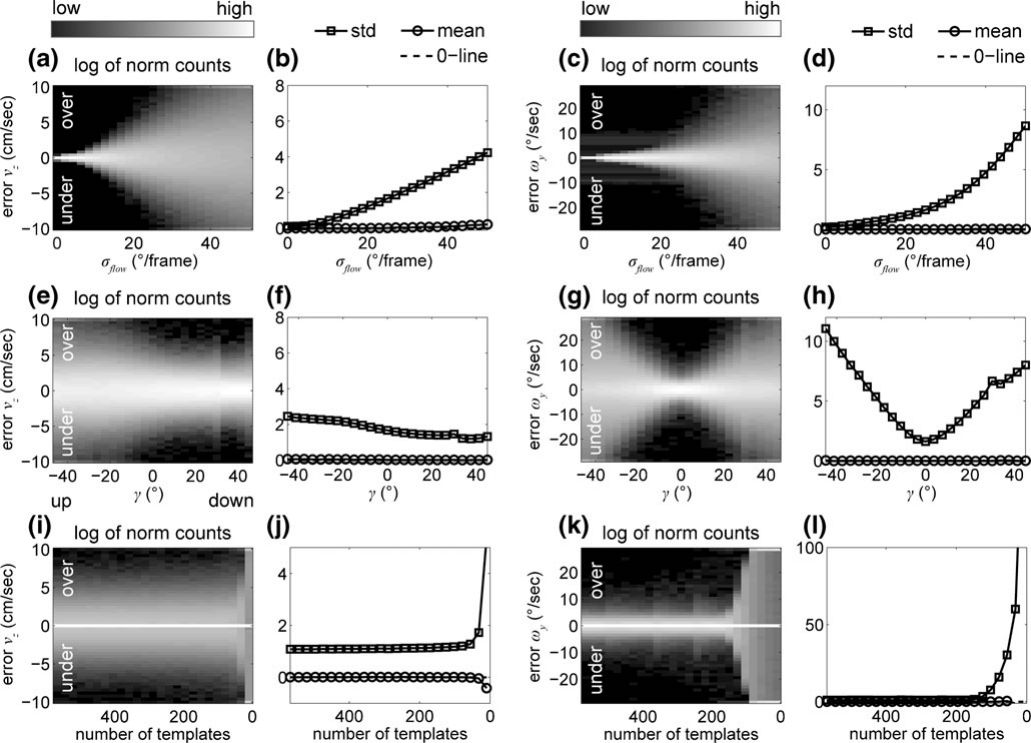
Errors for self-motion estimation while varying noise, tilt, or the number of flow templates. (**a**-**d**) shows the errors for varying Gaussian noise, (**e**-**h**) tilt angles, and (**i**-**l**) number of templates with *σ*_*flow*_ = 25 *°/frame* in the latter two cases. Normalized histograms (**a**, **c**, **e**, **g**, **i**, **k**) are individually computed for every parameter value. Normalized counts for each bin are log-enhanced and displayed in gray-values ranging from black to white encoding low and high counts. The standard deviation and mean value of errors are displayed in the curve plots (**b**, **d**, **f**, **h**, **j**, **l**). Note that the intervals for plots (**j**) and (**l**) are different from those in previous rows. Legends are printed atop of each column. The error statistics includes three trajectories

Simulated optic flow deviates from the model of visual image motion due to the Gaussian flow noise. Thus, we expect the template models’ estimates to have errors. Another source of errors is the finite sampling of the velocity spaces due to interpolation between sample points, although sampling intervals are adapted to the data. But what does the statistics of these errors look like? In three simulations we replay three recorded trajectories to the model and estimate self-motion from optic flow. Figure 4 depicts the error statistics for linear and rotational velocities that includes all three trajectories. Errors are defined as the estimate minus ground-truth value. Thus, errors of positive sign relate to an overestimation and errors of negative sign to an underestimation of velocity. For the first simulation of varying Gaussian flow noise, errors in the estimated linear velocity (Fig. 4(a) and (b)) and the rotational velocity (Fig. 4(c) and (d)) increase linear in terms of their standard deviation. Varying tilt shows an effect on the estimation (Fig. 4(e)-(h)). Downward tilts increase the length of azimuth and elevation vectors. This explains the decrease of error for conditions where gaze is directed down toward the ground. The error in rotational velocity estimates is symmetric around 0 ° tilt where it is smallest (Fig. 4(e)-(h)). The third simulation, that of varying the number of flow templates, shows large errors in the estimate of linear (Fig. 4(i) and (j)) and rotational velocity (Fig. 4(k) and (l)) for a small number of templates. This error drops to a lower nearly constant level around a number of 150 flow templates (Fig. 4(j) and (l)).

### Position errors for integrating velocity estimates

**Fig. 5:**
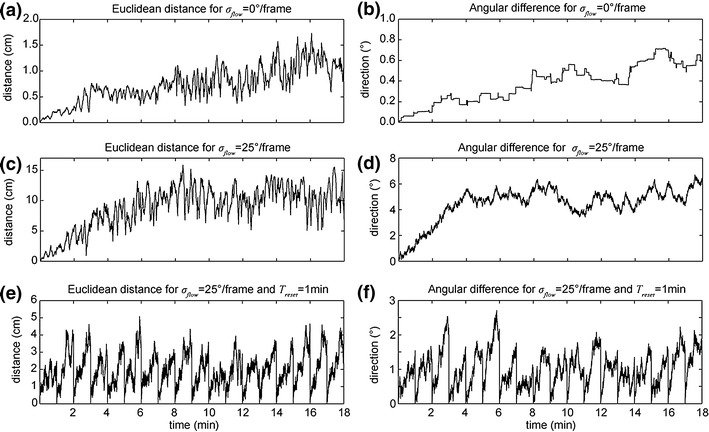
Position errors of temporally integrated estimates stay within (**a**) 3 cm Euclidean distance error and (**b**) 2 ° angle error for analytical image motion and within (**c**) 15 cm distance error and (**d**) 6 ° angle error for simulated optic flow that includes Gaussian noise with *σ*_*flow*_ = 25 °/frame. (**e** and **f**) Shows the distance and angle error for estimates from optic flow with Gaussian flow noise *σ*_*flow*_ = 25 °/frame where every *T*_*reset*_ = 1 min a reset to the ground-truth position and orientation happens. Note the difference in intervals for the y-axis between panels

After looking into the error statistics of velocity estimates we now study the position error that occurs if temporally integrating estimated velocities. As error measures we report the Euclidean distance and angle between the ground-truth and estimated position. Figure 5 shows mean distance and angle errors for analytical visual image motion (*σ*_*flow*_ = 0 °/frame) computed by using the three trajectories. Errors in distance are within 3 cm and errors in angle within 2 ° integrating over 18 min or 54,000 individual estimates (Fig. 5(a)-(b)). For the Gaussian flow noise of *σ*_*flow*_ = 25 *°/frame* mean error range within 15 cm for the distance and 6 ° for angles. These results look promising and suggest that integration of optic flow into a path integration system can be helpful.

### Grid scores for varying Gaussian flow noise, tilt angles, and number of templates

**Fig. 6:**
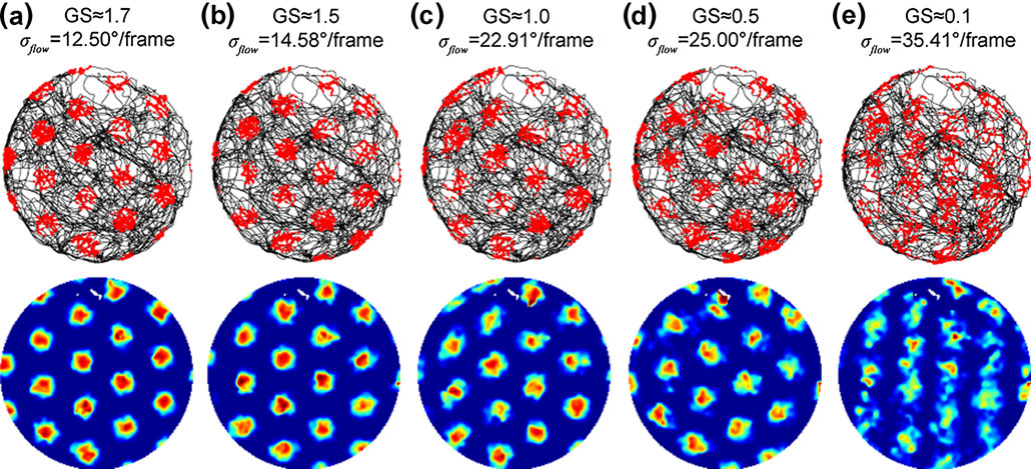
Examples for firing patterns of varying grid score (GS). The first row shows the different trajectories (*black traces*) with superimposed locations where a spike occurred (*red dots*). In the second row these spikes are registered in a 201 × 201 pixels image and are divided by the occupancy in these locations. Both registered spikes and occupancy are convolved with a squared Gaussian filter with nine pixels length and two pixels standard deviation. (**a**) A moderate amount of Gaussian flow noise (*σ*_*flow*_ = 12.5 °/frame) leads to a GS of 1.7. Increasing the amount of noise gives GS of 1.5 in (**b**), 1.0 in (**c**), 0.5 in (**d**), and 0.1 in (**e**). All values are for a reset interval of *T*_*reset*_ = 16.67 min and parameters of Gaussian flow noise *σ*_*flow*_ are given atop of each trajectory plot. The simulation uses the trajectory ‘Hafting_Fig2c_Trial1’ from Hafting et al. (2005). Note that the response patterns in the lower row are individually scaled to min (*blue*) and max (*red*) values of spikes per second. Regions plotted in white have never been visited, in this example the gap in direction “North”

What is the effect of the errors in the velocity estimates on grid cell firing patterns? Estimated velocities are integrated into the velocity controlled oscillatory interference model that was reviewed in Section 2.6. Grid cell firing patterns are shown in Fig. 6. To quantify the grid-likeness of these patterns a grid score (GS) measure is computed (Langston et al. 2010; their supplement material on page 11). This GS ranges within the interval [–2… + 2]. Intuitively, the GS is high if the same spike pattern matches itself for rotations of 60 ° and multiples of 60 ° (hexagonal grid) and the GS is low if the grid matches itself for rotations of 30 ° and multiples of 30 ° (e.g. a squared grid). We illustrate this GSs measure in Fig. 6 by showing examples of grids of different scores. Note that for this data 1.7 is the highest GS value that could be achieved.

For the spike pattern in Fig. 6(a) we estimated the grid distance from the data by clustering the spikes using the k-means algorithm (10 retrials). The mean between-cluster distance for direct neighboring vertices is approximately ≈ 39 cm 3 and the theoretical value is calculated as $G = 2/(\sqrt {3} \cdot f \cdot \beta )$ or $G = 2/(\sqrt {3} \cdot 7.38Hz \cdot 0.00385\sec /cm) \approx 41cm$.

**Fig. 7:**
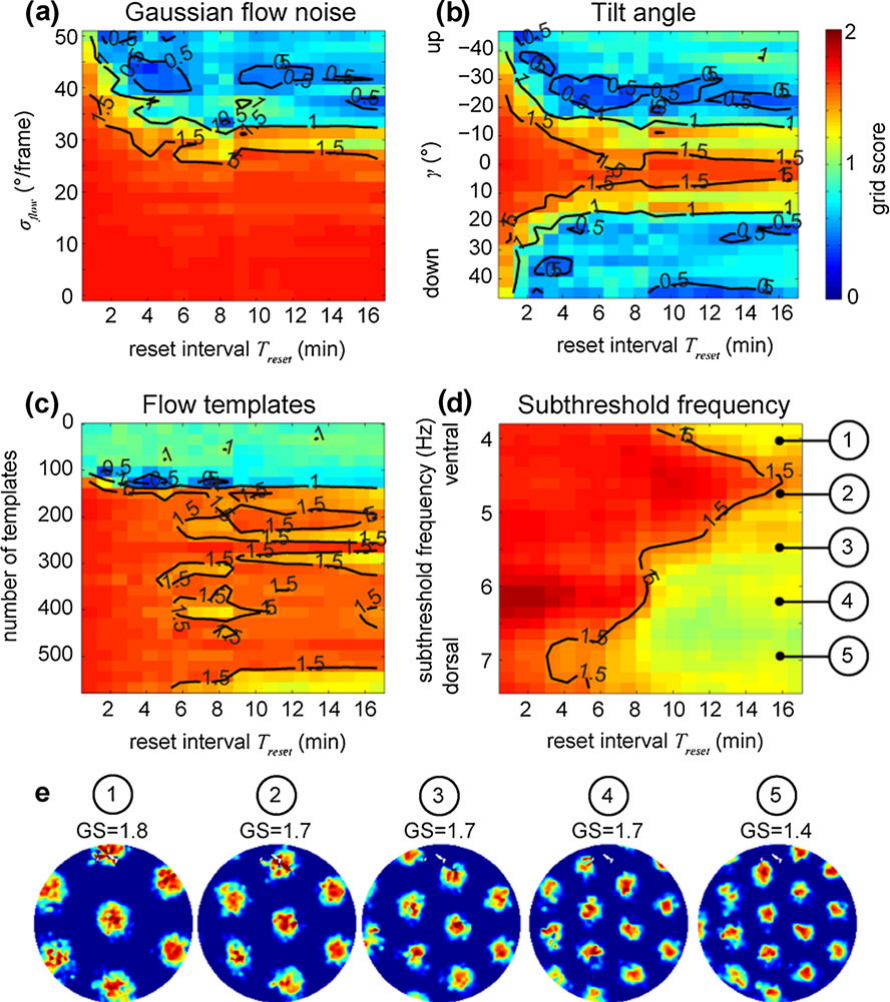
In most cases grid-cell firing could be achieved by integrating optic flow input when assuming a grid score (GS) greater than one. (**a**) Shows the GS in color (blue 0 and red 2) for different reset intervals (x-axis) and Gaussian flow noise (y-axis). A legend for the color code is printed on the right side. Black contour lines help to identify different regions with similar GS. Subsequent plots use the same color coding and show the GS for a varying tilt angle in (**b**), for a varying number of flow templates in (**c**), and for a varying frequency in (**d**). (**e**) Examples of firing patterns from the simulation in (**d**) as indicated by the number in circles for the trajectory ‘Hafting_Fig2c_Trial1’ from Hafting et al. (2005). Note that these grid scores differ from those shown in (**d**) that are mean values computed by using the three trajectories as noted in footnote ‘a’ in Table 1. For this plot we took the absolute value of the GS before computing the mean for different phases and trajectories

Does a reset mechanism that re-locates the rat with a certain periodicity *T*_*reset*_ help to improve the grid cell firing? Besides optic flow other cues, such as visual landmark cues, somatosensory cues, or vestibular cues are available to the rat. Therefore, a reset mechanism that uses these cues is included into the simulation. This mechanism validates the flow integrated position with the position estimates from other cues. In the simulation the velocity integrated position is corrected to its ground-truth position using the recorded trajectory. This correction takes place after each temporal period *T*_*reset*_ .^4^ In the simulation we vary this temporal period between 0.83 min and 16.67 min in steps of 0.83 min. This gives a 3D plot reporting GS over two varying parameters. Figure 7 shows these 3D plots, using a color code for the GS as third dimension. For varying Gaussian noise (y-axis) and varying reset intervals (x-axis) a transition between high GSs (>1) to GSs (<1) occurs for a standard deviation of ≈ 35 °/frame, largely independent of the reset interval *T*_*reset*_ (Fig. 7(a)). For a tilt angle *γ* ≈ 0 ° GSs are higher than 1.5 (Fig. 7(b)) mainly due to the low error in rotational velocity (compare with Fig. 4(g)). Grid scores for around 150 flow templates change rapidly from above one to below one (Fig. 7(c)). Different reset intervals have a small effect visible by the drop being aligned with the x-axis or reset interval.

Varying the frequency parameter of the oscillatory interference model influences the grid spacing. For instance, for *f* = 7.38 Hz the grid spacing is 41 cm and for *f* = 3.87 Hz the grid spacing is 77 cm. This varying grid spacing shows an effect coupled to the reset interval *T*_*reset*_ (Fig. 7(d)). For a reset interval above *T*_*reset*_ = 12 min and a frequency below 5.5 Hz the GS is ≈ 1. This corresponds to the upper right corner in the 2D plot in Fig. 7(d). For frequencies above 5.5 Hz and a reset interval above ≈ 9 min the GS is ≈ 1. Other regions have a GS > 1. Our interpretation is that for low frequencies only a few vertices—around seven—are marginally present and, thus, no full clear cell of a hexagonal grid, consisting of seven vertices, is formed for all trajectories (although it is in the example trajectory shown in Fig. 7(e) 1). This partial occurrence of less than seven vertices reduces the GS. For slightly higher frequencies ≈ 4.5 Hz one full cell of the hexagonal grid is present and, thus, in these cases a higher GS of ≈ 1.5 is achieved. For a further increased frequency of >5 Hz, multiple additional firing fields of the grid are present (Fig. 7(e) 3). All these firing fields have to be accurate and this reduces the GS to ≈ 1.

## Discussion

We investigated the role of optic flow available to rats and their possible influence on grid cell firing in a modeling study. Our model uses a spherical camera to simulate the large visual field of rats and models the sensed optic flow as analytical visual image motion with superimposed Gaussian noise. This sensed flow is compared to flow templates for linear and rotational velocities that are sampled in intervals according to the statistics of these velocities in recorded rat trajectories. Maximum responding templates and their neighbors are used to estimate linear and rotational velocity. These estimates are fed into the velocity controlled oscillator model to produce grid cell firing. Grid scores from this overall model using optic flow as input are above 1.5 even for Gaussian flow noise with a standard deviation up to 35 °/frame. In addition our model requires at least ≈ 100 flow templates to achieve grid scores above one (GS > 1). The tilt angle of the simulated spherical camera with respect to the ground affects the grid score. Overall our modeling study suggests optic flow as a possible input to grid cells beside other cues.

### Template models for self-motion estimation using optic flow

Various template models have been suggested for self-motion estimation from flow. Perrone (1992) suggested a template model that estimates, first, rotational velocities from the input flow and, second, subtracts the flow constructed by these estimated rotational velocities from the input to estimate the linear velocities of self-motion. Later, Perrone and Stone (1994) modified this model by setting up flow templates for common fixating self-motions for human locomotion. Lappe and Rauschecker (1993) used a subspace of flow constraints that is independent of the depth and rotational velocities. The remaining constraints that depend on linear velocity only are used to set up flow templates. In contrast to these models, we simplified the parameter space by restricting the rat’s body motion to curvilinear path motion that is composed of linear velocity parallel to the ground and a rotational velocity normal to the ground. This reduces the degrees of freedom from six to two that are estimated independently by two 1D spaces of flow templates: One for linear velocities and one for rotational velocities. Similar to Perrone’s (1992) template model, the accuracy of self-motion estimation in the presence of flow noise increases with an increasing number of samples, given that the noise for samples is independent (Perrone 1992; his Fig. 5). For that reason we did not include the number of sampled flow vectors as a parameter of our simulations.

Although sharing this behavioral similarity, our template model is conceptually different from prior ones. It uses a spherical camera model instead of a pinhole camera. It resolves the scaling invariance between linear velocity or speed and absolute depth by assuming points of flow sampled from a ground plane and knowing the distance and angle of the camera with respect to that plane. Our model extends prior ones by allowing for variable tilt of the camera that leaves the direction of self-motion unaffected. In our model a separation of estimating linear and rotational velocity is achieved by multiplying the flow equation by a vector orthogonal to the rotation induced flow (see Eq. (6)). This separation leads to a speed-up of simulation times and requires less storage—or fewer “template neurons”. Rather than testing all combinations of linear and rotational velocities that would lead to a squared complexity in match calculation and storage, we tested linear and rotational velocities separately. This required only linear complexity in the number of samples in each dimension. This “computational trick” was applied for the convenience of the simulations rather than assuming the same trick is being applied in the rat visual system. In sum, our model provides several extensions compared to prior template models.

### Models for grid cell firing integrate linear 2D velocities

Models for grid cell firing temporally integrate a 2D velocity signal (Burgess et al. 2007; Hasselmo 2008; Burak and Fiete 2009; Fuhs and Touretzky 2006; Mhatre et al. 2012). None of these models elaborate on the details of estimating this 2D velocity signal, other than assuming this signal being split into a head direction signal assumed to be provided by the head direction cell system and a linear speed signal assumed to be provided by the vestibular system. A realistic model would estimate the 2D velocity that in turn introduces errors that could be characterized by a noise model. However, noise is not assumed in the velocity signal in models of grid cell firing. Instead noise is assumed in the neural population of attractor models (Fuhs and Touretzky 2006; Burak and Fiete 2009) or in the phase of the oscillatory interference models (Burgess et al. 2007; Zilli et al. 2010).

In our study we elaborated on the modeling of the velocity signal, suggesting optic flow as an alternative cue besides the traditional view that velocities are supported by the vestibular system. This approach naturally leads to errors in the 2D velocity signal, as the velocity signal is estimated from optic flow. Our analysis of errors shows that most parameter settings in our model of self-motion estimation and optic flow allow for path integration that is accurate enough to produce the grid cell’s typical firing pattern.

### Many species use optic flow for navigation and depth estimation

Optic flow influences the behavior in many species. Pigeons bob their heads during locomotion, which is mainly visually driven. This bobbing supports the stabilization of vision during the period of forward movement. The forward movement is compensated by a backward motion of the head caused by the bird’s neck zeroing its retinal motion (Friedman 1975). Behavioral data shows that honeybees use image motion for navigation in a corridor (Srinivasan et al. 1991). Mongolian gerbils use motion parallax cues and looming cues to estimate distance between two platforms where their task is to jump from one platform to the other (Ellard et al. 1984). For humans, Gibson (1979; page 111 ff.) highlights the relevance of optic flow during aircraft landing and more generally during locomotion. Humans can judge heading from translational flow within the accuracy of one degree of visual angle (Warren et al. 1988). For flow including simulated or anticipated eye-movements the accuracy is within two degrees of visual angle (Warren and Hannon 1990). Although, the resolution of rat vision is poorer compared to that of humans we suggest that rats are able to register large flow field motion as generated by self-motion in a stationary environment. In turn, such flow fields encode the rat’s self-motion which motivates our hypothesis that rats might use optic flow among other cues.

Studies using macaque monkeys reveal the underlying neural mechanisms of optic flow processing and estimation of self-motion. Neurons in area MST show selectivity for the focus of expansion (Duffy and Wurtz 1995; Duffy 1998) and to spiral motion patterns induced by a superposition of forward motion and roll motion of the sensor (Graziano et al. 1994). The selectivity for the focus of expansion is shifted by eye-movements (Bradley et al. 1996). Studying the connectivity, these neurons in area MST integrate local planar motion signals over the entire visual field by spatially distributed and dynamic interactions (Yu et al. 2010). Furthermore, MST neurons responded stronger if monkeys had a visual steering task compared to passive viewing. This suggests that task driven activation can shape MST neuron responses (Page and Duffy 2007). The activation of MST neurons is influenced by objects moving independently in front of the background (Logan and Duffy 2005). An electrical stimulation of MST neurons during a visual navigation task affected monkey’s decision performance, typically leading to random decisions and these were amplified in the presence of eye movements. This suggests that area MST is involved in self-motion estimation with a correction for eye-movements (Britten and Wezel 2002). An overview of mechanism in area MST, its influence on steering, and the solution of the rotation problem—the segregation of translation and rotation—is given by Britten (2008). No analogue to MST cells has been reported for rats, so far. But especially monkey physiology suggests experimental conditions to probe the optic flow system. Large field motion stimuli of expansion or contraction with a shifted focus of expansion or laminar flows are typical probes (see Figs. 2 and 3). In our model we assumed cells tuned to these flows for the estimation of linear and rotational velocity.

### Optic flow cues might not be limited to influence only grid cells

Several cell types could profit from optic flow information. *Border cells* or boundary vector cells that respond as the animal is close to a border with or without wall (Burgess and O'Keefe 1996; Solstad et al. 2008; Lever et al. 2009; Barry et al. 2006) could receive input from hypothetical cells detecting self-motion patterns which are sensitive to different distributions of image motion speeds. In a related study we propose a model for boundary vector cell firing based on optic flow for rats in cages with and without walls (Raudies and Hasselmo 2012). In the presence of walls the challenge is to achieve a flow-based segmentation of walls from ground, as samples from these different surfaces play behaviorally different roles as we suggest. Flow samples from the ground allow for the estimation of linear velocity, and thus could facilitate grid cell firing. In contrast, flow samples from walls do not encode velocity above ground, rather they allow for a distance and direction estimate of walls if the velocity above ground is known. Thus, flow samples from the ground combined with flow samples from walls could facilitate the firing of boundary vector cells that fire dependent upon the boundaries of the environment. Segmentation of flow can also be used for the detection of physical drop-offs at the boundaries of an environment (which activate boundary vector cells). This is challenging in simulations if the rat is far away from the drop-off because in that case the flow close to the horizon at the drop-off has a small magnitude and the discontinuity in the flow between the edge of the drop-off and the background is hard to detect. In our related study we propose a mechanism for the detection of drop-offs and the conversion into distance estimates of the drop-off. This model builds upon the spherical model proposed here but is different in mechanisms and modeled cell populations. Together, our model of grid cell firing in this paper and the modeling of boundary vector cell firing in the related study support the hypothesis that the firing of these different cells might be facilitated by optic flow in a multi-modal integration with other cues.

*Head direction cells* (‘compass cells’) could update their direction specific firing in a 2D plane, e.g. for north, irrespective of the rat’s body or head position and rotation by integrating the estimated rotational velocity from optic flow (Taube et al. 1990). *Grid cells* (Hafting et al. 2005) and *place cells* (O’Keefe 1976) could fire based on the temporal integration of estimated linear and rotational velocity from optic flow, the latter is suggested by our model.

### Self-localization by cues other than optic flow

Experiments show the importance of vestibular and sensorimotor input to head direction cells which are part of a neural network for rat’s navigation. Head direction cells in the anterior thalamic nucleus depend on vestibular input, as lesioning the vestibular system drastically reduces the coherence in directional firing of these cells (Stackman and Taube 1997; their Figs. 1 and 4). Selectivity of these head direction cells could be generated by angular head velocity sensitive cells in the lateral mammillary nuclei (Stackman and Taube 1997; Basset and Taube 2001). Persistent directional firing of head direction cells in the anterior dorsal thalamic nucleus and postsubiculum was not maintained while the rat was moved in darkness into another room, whereas this transfer excludes sensorimotor and visual cues. This shows that vestibular input is not sufficient to maintain firing of head direction cells in all cases (Stackman et al. 2003).

Various scenarios show the importance of landmarks, either directly for behavior or for the firing of cell types. Over-trained rats in a Morris water maze with artificially generated water currents rely on landmarks rather than path integration of sensorimotor cues to find the hidden platform (Moghaddam and Bures 1997). *Head direction cells* (Taube et al. 1990) receive vestibular signals, visual motion signals, and signals about landmark locations. Furthermore, if visual motion and landmark cues contradict each other, often the head direction cells average signals from both cues (Blair and Sharp 1996). The firing patterns of *grid cells* rotate with landmarks, thus, grid cell firing is locked to visual landmarks (Hafting et al. 2005; their Fig. 4). If idiothetic cues are in conflict with landmarks, *place cells* rely largely on landmarks for small mismatches and hippocampus formed a new representation for large mismatches (Knierim et al. 1998). Another experiment changes the size of the cage while running the experiment. Grid cell firing showed a rescaling to the environment leading to a deformation of the hexagonal grid structure. This rescaling is slowly reduced with experiencing the newly sized environment and the grid cell firing pattern is restored to its intrinsic hexagonal structure. This suggests that rats rely on visual cues in familiar environments such as boundaries and grid cell firing gets deformed if these cues are shifted (Barry et al. 2007). In sum, the vestibular system and the visual landmarks play a major role in driving cells prior or close to entorhinal cortex and the grid cell system.

### Challenges to our flow-hypotheses

The results of our model simulations motivate two hypotheses. First, optic flow among other cues might contribute to the firing of grid cells. This hypothesis can be tested by using a virtual environment setup, similar to the one used by Harvey et al. (2009). In such a setup, the animal is running on a trackball and the corresponding video signal is shown on a display in front of the animal. In this case visual cues that lead to optic flow can be manipulated in two ways. Optic flow cues can be gradually removed while the animal is still provided with sensorimotor cues. According to our model less flow would lead to larger estimation errors for linear and rotational body velocities. For our multi-modal integration paradigm this would lead to a decrease in grid score for grid cells. Another manipulation is the update of the video being inconsistent with the movement of the animal on the trackball, e.g. instead of displaying a simulated forward motion showing a simulated backward motion. In this case we expect again a decrease in grid score as firing induced by optic flow becomes inconsistent with firing induced by e.g. sensorimotor signals. Our second hypothesis is that GS depends on the quality of the flow signal. From our simulation results we infer that for up to 30 °/frame standard deviation of Gaussian noise the grid score would still be about one for cells in the experiment, so they would still be classified as grid cells. This can be tested by superimposing noise in the video displayed to the rat in that again should affect the grid score of grid cells. Our hypothesis depends largely on the quality of optic flow that is available to the rat.

### Optic flow amongst other cues

Optic flow directly provides linear and rotational velocity of the rat’s motion. Note that our model only simulated body motion, not head or eye motion. These velocity estimates for body motion can be temporally integrated to result in self-position information with respect to a reference location. For the same position estimates linear and angular acceleration signals from the vestibular system would have to be temporally integrated twice. Temporal integration at two stages introduces an additional source of error accumulation in comparison to a single temporal integration used for optic flow. Other cues, like visual landmarks, provide a self-position signal without temporal integration and are in that sense at an advantage compared to optic flow. Also optic flow is not available in the dark or in environments that do not provide enough texture to pick up flow. In these cases it becomes very likely that sensorimotor signals and vestibular signals are used for path integration. On the other hand, visual input might cause the observed compression and expansion effects of grid cell firing fields as a result of expanding or shrinking the rat’s cage during the experiment. For instance, optic flow that is generated from an altered cage size would be different and can indicate an expansion or contraction. Optic flow can be used for path integration during the initial phase of exploration before landmarks are learned and the environment is mapped out. In sum, optic flow might be one cue in the multi-modal integration of cues that lead to the firing of grid cells in cases where flow is available by texture together with sufficient lighting.

## Footnotes

1 Note that these references use different definitions for their coordinate system.

2 As an alternative this signal *φ(t)* can be provided by the head direction system. Such a signal has been used in Eq. (4) in cases where the ground’s normal vector depends on head orientation. This is the case for non-zero tilt angles.

3 Several runs of the k-means algorithm can give slightly different values due to a randomized initialization of the initial cluster centers.

4 To avoid a correlation effect between reset times and specific velocity configurations in the data, we evaluate ten different phase values, the onset times of the reset mechanism with respect to time zero that are regularly distributed within the duration of two resets.
